# Micropropagation of *Ficus religiosa* L. via leaf explants and comparative evaluation of acetylcholinesterase inhibitory activity in the micropropagated and conventionally grown plants

**DOI:** 10.1007/s13205-013-0175-8

**Published:** 2013-10-04

**Authors:** Priyanka Siwach, Anita Rani Gill

**Affiliations:** Department of Biotechnology, Chaudhary Devi Lal University, Sirsa, Haryana India

**Keywords:** In vitro regeneration, Shoot multiplication, Clonal nature, RAPD markers, Acetylcholinesterase inhibitory activity, Alzheimer disease

## Abstract

A high-frequency, season-independent, in vitro regeneration of *Ficusreligiosa* was developed, followed by comparative acetylcholinesterase inhibitory (AChEI) activity assay of the in vitro raised and conventionally grown plants. The use of AChEI activity is the most accepted strategy for the treatment of Alzheimer disease. Fully expanded, mature leaves were cut into different segments to initiate the cultures. The middle section of the leaf in vertical orientation with cut portion inserted inside the medium was found most suitable for direct shoot regeneration. Leaf explants responded with nearly consistent frequency (60–66.67 %) throughout the year. To obtain high frequency response with enhanced shoot multiplication rate, 32 plant growth regulator regimes were screened amongst which benzylaminopurine at 5.0 mg/l was found most suitable, yielding 100 % response and maximum number of shoots per explant (7.93); same concentration was also most supportive for repeated multiplication (6.53 shoots). The quality of the shoots and multiplication rate could be significantly enhanced (24.35 shoots) when adenine sulphate, glutamine and phloroglucinol, in an optimised concentration, were additionally supplemented. The clonal nature of the micropropagated plants was confirmed by random amplified polymorphic DNA analysis. A comparative analysis of AChEI activity was carried out amongst the methanolic extracts of stem segments of the mother plant, randomly selected seedlings of different age (4 and 6 months old) of the same mother plant and randomly selected micropropagated plants of different age (3 and 6 months age). The mother plant sample showed effective AChEI activity, with IC_50_ of 66.46 μg/ml while seedlings, of different age groups, performed poorly (6-month-old seedlings, Se-1_6M_, yielded IC_50_ of 20,538.46 μg/ml, while two randomly selected 4 months’ aged seedlings, Se-2_4M_ and Se-3_4M_ exhibited IC_50_ of 19,341.03 and 24,281.70 μg/ml). On the other hand, various micropropagated plants, 2 of 3 months (MiP-1_3M_, MiP-2_3M_) and 2 of 6 months (MiP-3_6M_ and MiP-4_6M_) age behaved like the mother plant, exhibiting IC_50_ values of 71.87, 72.91, 67.65 and 69.65 μg/ml, respectively.

## Introduction

*Ficus religiosa*, the Sacred Fig or Bo-Tree, belonging to family Moraceae, is a large heavily branched tree with long petiolated, heart-shaped leaves. The tree is native to India and Nepal where it has great ethano-medicinal and religious importance since times immemorial. Different parts of the tree render applications for more than 50 disorders, which are well documented in Ayurveda, the indigenous Indian medicine system (Singh et al. 2011). Many of these have been confirmed by various pharmacological studies (Kirana et al. 2009; Pandit et al. 2010). One of the most affective and popular use of *F*. *religiosa* is in the treatment of cognitive decline, improving memory and related central nervous system disorders (Singh and Goel 2009). A number of traditional practitioners in north India prepare specific herbal formulations from stem bark of *F. religiosa* for treatment of memory loss and various neuro-degenerative disorders (Personal communication). The scientific basis for this was revealed by Vinutha et al. (2007), who while analysing the methanolic stem bark extract of *F. religiosa*, found potent acetylcholinesterase inhibitory (AChEI) activity associated with it. The latter is the most accepted strategy for the treatment of Alzheimer disease (AD) and other related diseases (Bertaccini 1982); the inhibitors prolong the half-life of acetylcholine through inhibition of acetylcholinesterase (AChE) (Darvesh et al. 2003). Since, the present day drugs for AD (tacrine, donepezil, rivastigime and galanthamine) are suffering with short-half-lives and/or unfavourable side effects such as hepatotoxicity, low bioavailability, adverse cholinergic side effects and a narrow therapeutic window (Sancheti et al. 2009), *F*. *religiosa* is being looked upon as a potential source of a new drug for AD. The phytochemical analysis of bark and stem of *F. religiosa* has led to the isolation of phytosterols, amino acids, furanocoumarins, phenolic components, flavonoids, saponins and acid detergent fibres (Ambike and Rao 1967; Swami et al. 1989; Swami and Bisht 1996); however, those responsible for AChEI activity are yet to be identified. There is a need to characterise and exploit the AChEI activity found in the stem bark of *F*. *religiosa* at the commercial level. Conventionally, the tree is propagated by seeds, which remain viable for a few months and the plants produced are not true to types. The vegetative propagation by cutting is not efficient under varied climatic conditions. The nature and amount of secondary metabolites in different parts of the plants is greatly affected by the environmental condition and so naturally propagated plants are unable to yield consistent yield of secondary metabolites throughout the year (Gurel et al. 2011; Tamara et al. 2011). Micropropagation is a proven method for large-scale production of true-to-type medicinal plants capable of yielding desired plant-derived pharmaceuticals with consistent quality and amount (Pattnaik and Chand 1996; Jiang et al. 2012). In vitro propagation methods also offer the opportunity to correlate the secondary metabolite production with several parameters like nutritional and hormonal composition of nutrient medium, growth conditions, duration of culture, etc., and so are better for production of plants for commercial, pharmaceutical applications (Gurel et al. 2011).

Some previous work on in vitro propagation of *F. religiosa* L. using nodal segments as explants has been carried out by Jaiswal and Narayan (1985), Deshpande et al. (1998), and Hassan et al. (2009). In an attempt to further improve the micropropagation protocol (Siwach and Gill 2011), it was observed that response of nodal segments as well as frequency of contamination under in vitro conditions was strongly influenced by the season of explants collection, restricting the culture initiation experiment to a particular time-period of the year (Siwach et al. 2011). Similar observations were also observed with apical shoot explants (data not shown). The seasonal influence on the establishment and response of in vitro cultures of perennial trees is due to their periodic development and is one of the major hurdles towards commercialisation of micropropagation for these plants as it cannot be overcome by environmental or nutritional manipulations (Siril and Dhar 1997; McCown 2000).

Therefore, a need was felt to formulate a commercially viable micropropagation protocol for *F. religiosa* L., which would be beyond the seasonal constraints. Axillary and apical meristematic cells (on the nodal and apical explants respectively), of the perennial trees, are genetically directed to divide actively during the active growth season, and this periodic growth direction may not be there with the differentiated tissues like leaves. There exists one study reporting the use of leaves as explants for in vitro propagation of *F*. *religiosa* (Narayan and Jaiswal 1986). However, the study pertains to indirect shoot organogenesis from leaves through the callus phase and does not discuss about the seasonal influence on the in vitro response of leaves’ explants. So during the present study, experiments were initiated with fully expanded mature leaf segments to optimise season independent protocol for *F*. *religiosa*. It was planned to go specifically for direct shoot regeneration followed by confirming the clonal nature of plants by randomly amplified polymorphic DNA (RAPD) based molecular analysis. The latter has been reported as a reliable method for monitoring the genetic stability of micropropagated plants in many species (Khan and Spoor 2001). To explore the pharmaceutical potential of micropropagated plants, present study was extended to carry out comparative AChE inhibitory activity assay among the stem tissue of randomly selected micropropagated plants, randomly selected seedlings of the same mother plant and mother plant itself.

## Materials and methods

### Plant materials

Fully grown, healthy looking leaves were excised from third to sixth node from the tip of a healthy branch of a 45–50-year-old tree of *F. religiosa* L., selected as the mother plant (MP) for the present study, growing near the campus area of Chaudhary Devi Lal University, Sirsa, Haryana, India. The leaves were kept for 15 min under running tap water to remove the traces of dust and were surface sterilised as described earlier (Siwach and Gill 2011). The surface sterilised leaves were used as explants for direct shoot regeneration in the present study.

Seeds of the same mother plant were also sown in the month of March (seeds are available during months of March to June in North India) under greenhouse conditions and were properly taken care of. Among various seedlings so obtained, three seedlings of different ages were randomly selected. One seedling was of 6 months age (named as Se-1_6m_) while other two were of 4 months age (named as Se-2_4M_ and Se-3_4M_). These three seedlings were used for comparison with micropropagated plants in different analysis, reported below.

### Effect of explant orientation on shoot organogenesis

The surface sterilised leaves, as obtained above, were cut into three sections—proximal section with petiole, middle section and distal section with leaf tip. Each section was further cultured in three orientations-cut portion inserted in medium, adaxial surface touching the medium and abaxial surface touching the medium. Murashige and Skoog’s medium (1962), (MS) supplemented with 1.0 mg/l benzylaminopurine (BAP) was used for this experiment. The number of explants initiating shoot buds (percentage of response) and the average number of shoot buds per explant was recorded after 28 days of culture. The explants with sprouted shoot buds/shoots were shifted to the same medium as such within an interval of 30 days so as to score the number of shoots and the length of shoots after 60 days of initial culture (Table 1). Every treatment contained ten replicates and the experiment was repeated thrice. These experiments were carried out during January–February, 2009.

**Table 1:** Effect of different orientations of various cut leaf sections of *Ficus religiosa* L. on shoot organogenesis (cultured on MS medium having 1.0 mg/l BAP)

Cut leaf section	Orientation	Response (%)	No. of shoot buds^A^	No. of shoots^B^	Length of shoots^B^
Proximal section with petiole	Cut portion inserted vertically in medium	40.00^abc^ ± 9.09	3.33^b^ ± 0.15	3.40^b^ ± 0.18	1.97^bc^ ± 0.18
	Adaxial side touching the medium	36.67^abc^ ± 8.95	2.80^c^ ± 0.11	2.53^c^ ± 0.11	2.13^bc^ ± 0.19
	Abaxial side touching the medium	33.33^bc^ ± 8.75	1.33^e^ ± 0.11	1.47^d^ ± 0.13	1.67^c^ ± 0.09
Middle section	Cut portion inserted vertically in medium	63.33^a^ ± 8.95	4.83^a^ ± 0.18	4.57^a^ ± 0.13	2.67^a^ ± 0.19
	Adaxial side touching the medium	60.00^ab^ ± 9.09	3.47^b^ ± 0.21	3.37^b^ ± 0.19	2.13^bc^ ± 0.12
	Abaxial side touching the medium	56.67^abc^ ± 9.20	3.23^bc^ ± 0.20	2.33^c^ ± 0.11	2.03^bc^ ± 00.11
Distal section with leaf tip	Cut portion inserted vertically in medium	36.67^abc^ ± 8.95	2.23^d^ ± 0.18	2.17^c^ ± 0.14	2.47^ab^ ± 0.17
	Adaxial side touching the medium	33.33^bc^ ± 8.75	1.53^e^ ± 0.21	1.17^d^ ± 0.14	2.17^bc^ ± 0.18
	Abaxial side touching the medium	30.00^c^ ± 8.50	1.33^e^ ± 0.09	1.17^d^ ± 0.08	2.13^bc^ ± 0.17

Data are means from 10 replicates ± SE and those representing similar letter in the appropriate column are not significantly different (ANOVA, *P* ≤ 0.05), (groupings applying to whole table)

^A^Observed after 4 weeks of culture

^B^Observed after 60 days of initial culture

Using the most suitable orientation of the leaf segments as obtained from above experiment, following two studies were carried out in parallel.

#### Effect of season of explant collection on percentage of response of explants

The cut leaf segments, in the most suitable orientation as deduced from above experiment, were cultured every month from March 2009–February 2010. The MS basal medium supplemented with 1.0 mg/l BAP was employed for this study. There were 20 replicates for each month and the observations were recorded after 4 weeks of culture.

#### Effect of plant growth regulators on direct shoot regeneration

It was carried out during the period of March–July, 2009. The cut leaf sections in most suitable orientation as obtained above were inoculated on MS medium supplemented with various concentrations and combinations of BAP, thidiazuron (TDZ) and indole acetic acid (IAA). The observations were made for shoot organogenesis as reported above (Table 2). For each of these treatments, 20 explants were used and the experiment was repeated thrice.

**Table 2:** Effect of various concentrations and combinations of BAP, TDZ and IAA on shoot organogenesis from leaf explants

PGR (mg/l)			Response (%)	No. of shoot buds	No. of shoots^B^	Length of shoots (cm)
BAP	TDZ	IAA				
1.0	–	–	63.33^bcdefg^ ± 8.95	4.80^fg^ ± 0.23	4.53^d^ ± 0.16	2.67^abcde^ ± 0.16
1.5	–	–	60.0^cdefgh^ ± 9.09	3.93^hijk^ ± 0.33	4.57^d^ ± 0.18	2.47^defg^ ± 0.09
2.0	–	–	66.67^bcdef^ ± 8.75	3.93^hijk^ ± 0.28	4.33^de^ ± 0.24	2.23^efgh^ ± 0.12
2.5	–	–	66.67^bcdef^ ± 8.75	3.20^kl^ ± 0.25	4.07^de^ ± 0.29	2.07^ghi^ ± 0.14
3.0	–	–	73.3^bcde^ ± 8.21	3.73^ijkl^ ± 0.35	2.53^jk^ ± 0.22	2.50^cdefg^ ± 0.14
3.5	–	–	76.67^abcd^ ± 7.85	3.93^hijk^ ± 0.41	3.13^hi^ ± 0.21	2.53^bcdef^ ± 0.11
4.0	–	–	80.0^abc^ ± 7.42	4.23^ghi^ ± 0.31	4.20^de^ ± 0.29	2.33^defgh^ ± 0.18
4.5	–	–	83.33^abc^ ± 6.92	4.17^ghij^ ± 0.19	4.47^d^ ± 0.19	2.93^abc^ ± 0.15
5.0	–	–	100.0^a^ ± 0	5.83^e^ ± 0.19	7.93^a^ ± 0.20	2.97^ab^ ± 0.13
5.5	–	–	90.0^ab^ ± 5.57	4.13^ghij^ ± 0.31	3.23^ghi^ ± 0.08	2.20^fgh^ ± 0.10
6.0	–	–	63.33^bcdefg^ ± 8.95	2.13^n^ ± 0.08	1.17^m^ ± 0.07	2.17^fgh^ ± 0.09
1.0	–	0.5	43.33^fghij^ ± 9.20	3.70^ijkl^ ± 0.22	7.27^b^ ± 0.23	2.77^abcd^ ± 0.17
1.5	–	0.5	40.0^fghij^ ± 9.09	3.23^kl^ ± 0.10	3.47^fgh^ ± 0.19	3.07^a^ ± 0.15
2.0	–	0.5	36.67^ghij^ ± 8.95	3.43^ijkl^ ± 0.12	3.97^def^ ± 0.21	2.43^defg^ ± 0.11
2.5	–	0.5	36.67^ghij^ ± 8.95	3.07^lm^ ± 0.18	3.33^ghi^ ± 0.22	1.67^hijk^ ± 0.15
3.0	–	0.5	40.0^fghij^ ± 9.09	3.33^jkl^ ± 0.23	3.77^efg^ ± 0.21	1.57^ijk^ ± 0.18
3.5	–	0.5	36.67^ghij^ ± 8.95	3.33^jkl^ ± 0.22	2.77^ij^ ± 0.27	1.43^jkl^ ± 0.16
4.0	–	0.5	33.33^hij^ ± 8.75	3.93^hijk^ ± 0.12	2.10^k^ ± 0.19	2.57^bcdef^ ± 0.18
4.5	–	0.5	46.67^efghij^ ± 9.26	3.78^ijkl^ ± 0.20	4.30^de^ ± 0.26	2.97^ab^ ± 0.20
5.0	–	0.5	63.33^bcdefg^ ± 8.95	4.67^gh^ ± 0.15	5.53^c^ ± 0.32	2.67^abcde^ ± 0.27
5.5	–	0.5	50.0^defghi^ ± 9.28	2.23^n^ ± 0.09	3.13^hi^ ± 0.18	2.13^fgh^ ± 0.13
6.0	–	0.5	36.67^ghij^ ± 8.95	2.13^n^ ± 0.08	1.07^m^ ± 0.08	1.97^hij^ ± 0.20
–	1.0	–	63.33^bcdefg^ ± 8.95	5.50^ef^ ± 0.16	2.77^ij^ ± 0.23	1.07^kl^ ± 0.05
–	1.5	–	60.0^cdefgh^ ± 9.09	4.83^fg^ ± 0.25	2.33^jk^ ± 0.22	1.37^jkl^ ± 0.16
–	2.0	–	43.33^fghij^ ± 9.20	2.43^mn^ ± 0.41^A^	2.10^k^ ± 0.13	1.33^jkl^ ± 0.14
–	2.5	–	33.33^hij^ ± 8.75	2.33^mn^ ± 0.22^A^	2.03^kl^ ± 0.19	1.57^ijk^ ± 0.14
–	3.0	–	30.33^ij^ ± 8.51	1.97^n^ ± 0.22^A^	1.83^l^ ± 0.19	1.47^jkl^ ± 0.14
–	1.0	0.5	26.67^ij^ ± 8.21	10.17^b^ ± 0.39^A^	0	–
–	1.5	0.5	33.33^hij^ ± 8.75	12.33^a^ ± 0.36^A^	0	–
–	2.0	0.5	30.0^ij^ ± 8.51	9.30^c^ ± 0.36^A^	0	–
–	2.5	0.5	23.33^ij^ ± 7.85	8.87^c^ ± 0.27^A^	0	–
–	3.0	0.5	20.0^ij^ ± 7.43	7.07^d^ ± 0.17^A^	0	–

Data are means from 30 replicates ± SE and those representing similar letter in the appropriate column are not significantly different (ANOVA, *P* ≤ 0.05), (groupings applying to whole table)

^A^Extensive callus formation, ^B^observed after 60 days of culture

### Shoot multiplication during sub-culturing

The shoots regenerated from leaf explant were excised from the explant surface and were cultured in cluster of 2–3 shoots on six different medium. MS medium having most suitable PGR regime deduced from above study was taken as control. Two more concentrations of the selected PGR, lower than that in control, were also screened. These three media were further modified with specific combination of three additives—glutamine (200 mg/l), adenine sulphate (ADS) (150 mg/l) and phloroglucinol (100 mg/l), which was earlier reported to support the shoot proliferation during repeated sub-culturing (Siwach and Gill 2011). Observations were recorded after 35 days of culture (Table 3). For each of the above treatment, 20 replicates were used and the experiment was repeated thrice.

**Table 3:** Effect of different concentrations of BAP, with or without optimised additives, on shoot multiplication as well as quality of shoots (observations recorded after 5 weeks of culture)

BAP (mg/l)	Medium additives	No. of shoots	Length of shoots (cm)	Comments^A^
5.0 (control)	No additive	6.53^d^ ± 0.14	2.97^ab^ ± 0.13	−−
2.5	No additive	4.07^e^ ± 0.29	2.07^c^ ± 0.14	−−
1.0	No additive	4.53^e^ ± 0.16	2.67^b^ ± 0.16	−−
5.0	With optimised additives^B^	24.37^a^ ± 0.34	3.17^a^ ± 0.15	+++
2.5	With optimised additives^B^	17.17^b^ ± 0.09	2.23^c^ ± 0.13	+++
1.0	With optimised additives^B^	15.03^c^ ± 0.21	2.17^c^ ± 0.08	+++

Data are means from 30 replicates ± SE and those representing similar letter in the appropriate columns are not significantly different (ANOVA, *P* ≤ 0.05) (groupings applying to whole table)

^A^−−(Chlorosis, leaf fall), +++ (no chlorosis, no leaf fall, increased vigour of shoots)

^B^200 mg/l glutamine+ 150 mg/l adenine sulphate+ 100 mg/l phloroglucinol

### In vitro rooting, acclimatisation and transplantation

Individual shoots were subjected to in vitro rooting, acclimatisation and transplantation as reported previously (Siwach and Gill 2011).

### Culture conditions

MS medium, modified with different growth regulators as per requirement as reported above, was supplemented with 3 % (w/v) sucrose and solidified with 0.8 % (w/v) agar. The pH of medium was adjusted to 5.8 using 0.1 N NaOH or 0.1 N HCl, before autoclaving at 15 kg/cm^2^ and 121 °C for 20 min. The cultures were maintained at 25 ± 2 °C, at a photoperiod of 16 h (80 μmol m^−2^ s^−1^) in a culture room.

### Checking the clonal fidelity of micropropagated plants

Six micropropagated plants, of different age (1, 2, 4, 5, 6 and 7 months aged, after transplantation) were randomly selected for this. Three seedlings-Se-1_6M_, Se-2_4M_ and Se-3_4M_, discussed above in the “Plant material” sub-section, were also selected to check their clonal fidelity. Mother plant sample was taken as reference sample. Young leaves were excised from the above listed ten samples and were subjected to DNA isolation using Cetyl trimethyl ammonium bromide (CTAB) method (Sanghai-Maroof et al. 1984). Qualitative and quantitative assessment of total genomic DNA was carried out by spectrophotometer as well as agarose gel electrophoresis, followed by DNA purification.

Twenty RAPD primers, procured from Bangalore Genie Pvt. Ltd., were used for amplification (Table 4). Polymerase Chain Reaction (PCR) was carried out in a total volume of 15 μl with each reaction tube comprising of 8.1 μl of PCR water, 1.5 μl of PCR buffer containing MgCl_2_ (10X), 2.4 μl of dNTPs mixture (5 mM), 1.2 μl of primers (10 μM), 1.5 μl (10 ng/μl) of template DNA, and 0.3 μl of *Taq* DNA polymerase. Amplification was carried out using Bio-Rad thermal-cycler and was run for 35 cycles; each cycle consisting of a denaturation step at 94 °C (1 min), a primer annealing step at 36 °C (1 min) followed by amplification at 72 °C (3 min). Amplified products were loaded on 1.5 % agarose gel along with 100 bp ladder (GE Healthcare Life Sciences) and electrophoresis was carried out at 100 V. Gel was stained with 25 μg/ml ethidium bromide and photographed on a Gel Documentation Polaroid system (Bio-Rad).

**Table 4:** Amplification profile obtained with selected 20 RAPD primers, during the present study

Sr no.	Primer sequence	Band size of amplified products (bp)				
		Mother plant	Micropropagated plants	Se-1_6M_	Se-2_4M_	Se-3_4M_
1	CAGGCCCTTC	500	500	500, 670^A^	500	500, 600^A^
2	TGCCGAGCTG	1,200, 700	1,200, 700	700, 840^A^	700	700
3	AGTCAGCCAC	500	500	500	500	500
4	AATCGGGCTG	200, 300, 400, 500	200, 300, 400, 500	200, 300, 400, 500	200, 300, 400, 500	200, 300, 400, 500
5	AGGGGTCTTG	NA	NA	NA	NA	NA
6	GGTCCCTGAC	NA	NA	NA	NA	NA
7	GAAACGGGTG	NA	NA	NA	NA	NA
8	GTGACGTAGG	NRA	NRA	NRA	NRA	NRA
9	GGGTTTCGCC	240, 340, 440	240, 340, 440	300^A^, 590^A^, 610^A^	240, 340, 440, 590^A^, 610^A^	240, 340, 440, 590^A^, 610^A^
10	GTGATCGCAG	NRA	NRA	NRA	NRA	NRA
11	TAATCGGCGT	260, 350, 410, 600, 700	260, 350, 410, 600, 700	150^A^, 260, 350, 410, 600, 700, 900^A^	350, 410, 600, 700	150^A^, 260, 350, 410, 600, 700
12	TCGGCGATAG	280, 370, 480	280, 370, 480	280, 370, 480	280, 370, 480	280, 370, 480
13	CAGCACCCAC	280, 340, 500, 520, 600	280, 340, 500, 520, 600	280, 340, 500, 520, 600	280, 340, 500, 520, 600	280, 340, 500, 520, 600
14	TCTGTGCTGG	170, 380, 480, 690, 790	170, 380, 480, 690, 790	170, 380, 480, 690, 790	170, 380, 480, 690, 790	170, 380, 480, 690, 790
15	TTCCAAACCC	NA	NA	NA	NA	NA
16	TGCCATCGAA	NA	NA	NA	NA	NA
17	GGCGGCTTGT	300, 400	300, 400	300, 400	300, 400	300, 400
18	ATTGACCGT	550	550	550	550	550
19	CAAACGTCGG	300, 550, 700	300, 550, 700	300, 550, 700	300, 550, 700	300, 550, 700
20	GTTGCGATCC	420, 580, 640	420, 580, 640	420, 580, 640	420, 580, 640	420, 580, 640

*NA* No amplification, *N-RA* non-reproducible band pattern

^A^Polymorphic bands

### Sample preparation for AChE inhibitory activity assay

**Fig. 1:**
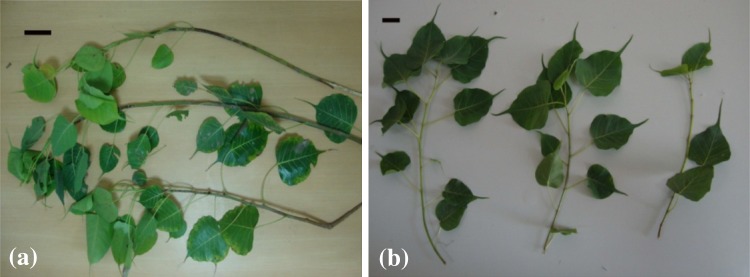
Source of stem tissue (for methanolic extract preparation). **a** Young branches of mother plant (of 45–50 year age), *bar* 10 cm; **b** young branches of seedlings/micropropagated plants (of age 3–6 months), *bar* 2.5 cm

Mother plant sample (MP) was taken as reference for this. Four micropropagated plants of different age (two plants after 3 months of transplantation—MiP-1_3M_ and MiP-2_3M_, and two plants after 6 months of transplantation—MiP-3_6M_ and MiP-4_6M_) were randomly selected. Three seedlings—Se-1_6M_, Se-2_4M_ and Se-3_4M_ were also taken for AChEI activity assay. The MP selected was of 45–50 years old, the age when higher secondary metabolites are reported to be present in stem bark. On the other hand, the other plants selected were of 3–6 months age as reported above. So to avoid the huge difference in the development stage of these plants, young, fresh, thin branches of mother plant was taken as sample (Fig. 1a) instead of stem bark or older branches. Likewise for seedlings and micropropagated plants also, young branches were excised (Fig. 1b). After excision, young branches were defoliated and cut into small-sized pieces followed by thorough washing with sterilised double distilled water. These were then dried in an oven at 32 °C. The dried pieces were finely ground to a fine powder using mixer grinder. The 15 g of dried fine powder of each sample was macerated separately with HPLC (high performance liquid chromatography) grade methanol (75 ml) at room temperature for 24 h. The extracts were obtained by filtration using Whatman No. 1 filter paper and concentrated to dryness. Working concentrations of methanolic extracts of each of the eight samples were prepared by completely dissolving the dried extract in methanol in ratio 1:1 to have stock solution of strength 1 mg/ml.

### AChE inhibitory activity assay

For each of the eight samples, five dilutions of 20, 40, 60, 80 and 100 μg/ml concentration were prepared. The assay for measuring AChEI activity was modified from the method described by Ellman et al. (1961) and Ingkaninan et al. (2000). Briefly, 4 μl of 3 mM dithiobis nitrobenzoic acid (DTNB), 20 μl of 15 mM acetylthiocholine iodide (ATCI), 130 μl Tris–HCl and 20 μl of each diluted sample was taken and added to the wells of microplate, followed by 50 μl of 0.28 U/ml AChE enzyme. The microplate was then read at 405 nm every 5 s for 2 min by a CERES micoplate reader (Spectra Max Plus 384, Molecular Devices, SoftMax Pro 5 S·No. SMP500-16135-DPVW). Mean absorbance per minute (*A*) was calculated for each dilution of different samples. Each plate had one blank well and one control well also. Percentage of inhibition of enzyme activity by a given concentration of sample was calculated by using the formula:

$$\text{Per cent inhibition}\;=\;\left[\left(A_{\text{control}}-A_{\text{extract}}\right)/A_{\text{control}}\right]\times 100$$

The percentage of inhibition obtained above was plotted vs. corresponding concentration of the extract of each sample. There were three replicates for each diluted concentration. The experiment was repeated for two more times.

### Calculating the IC_50_ value of each sample

The obtained graph of each sample fitted perfectly in Logarithmic curve shape and was subjected to regression analysis using logarithmic equation. The IC_50_ value (i.e. the concentration at which the enzyme activity is inhibited by 50 %) was calculated with the obtained equation.

### Statistical analysis

The experiments were set up in completely randomised design (CRD). The data were analysed by Analysis of Variance (ANOVA) followed by Duncan multiple range test (DMRT). Data analysis was carried out by using SPSS version 18.

## Results

### Effect of explant orientation on shoot organogenesis

**Fig. 2:**
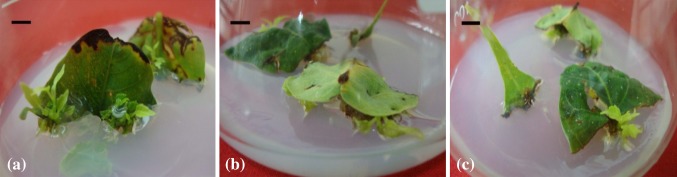
Induction of shoot buds/shoots from middle section of leaf cultured in; **a** vertical orientation with one cut end inserted inside the medium, *bar* 0.4 cm; **b** horizontal orientation with adaxial surface touching medium, *bar* 0.7 cm; **c** horizontal orientation with abaxial surface touching the medium, *bar* 0.7 cm

The leaf sections, in all the orientations, exhibited shoot-buds formation after 17–20 days of culture. Of the three types of cut leaf sections, middle section of the leaf performed better than the proximal or distal sections in each orientation (Table 1). Middle section responded with maximum frequency (63.33 %) in vertical orientation with cut portion inserted inside the medium, which was not significantly different to that obtained with horizontal orientation with adaxial surface (60.00 %) or abaxial surface (56.67 %) touching the medium (Table 1). Significantly higher number of shoots buds (4.83) which proliferated into equally higher number of shoots (4.57) with considerably more lengths (2.67 cm) was obtained with middle section of leaf in vertical orientation (Table 1). Distal sections and proximal sections responded poorly (40 % or less than it) in all the three orientations. Regardless of the nature and orientation, shoots originated from the cut ends of leaf segments having direct contact with the medium (Fig. 2a–c).

Middle section of the leaves with cut portion inside the medium was selected for the subsequent studies and here onwards it will be referred as explant/leaf explant.

### Effect of season on shoot organogenesis from leaf explant

**Fig. 3:**
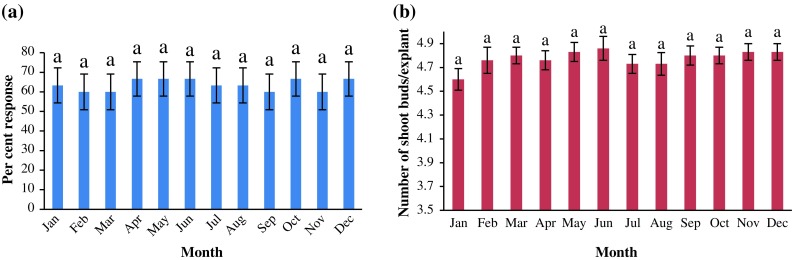
Effect of month of explant collection on; **a** percent response of explant; **b** average number of shoot buds per leaf explant (observed after 28 days of culture)

With the same medium condition, percentage of response of leaf explant varied from a maximum of 66.67 % (during the months of April, May, June, October and December) to a minimum of 60 % (during the months of February, March, September, November), the difference being statistically non-significant (Fig. 3a). Further, the maximum number of shoot buds per explant (4.86) was obtained for the month of June and this was not significantly different to that obtained during rest of the months (Fig. 3b).

### Effect of plant growth regulators on direct shoot regeneration

Leaf explants responded differently to different concentrations of BAP and TDZ. With increase in concentrations of BAP from 1.0 up to 5.0 mg/l, a continuous increase in percentage of response was observed, maximum being on 5.0 mg/l (100 %), afterwards the frequency decreased. On the other hand, with increase in concentration of TDZ from 1.0 to 3.0 mg/l, percentage of response continuously decreased; maximum was observed on 1.0 mg/l (63.33 %) (Table 2). Further, beyond 1.5 mg/l of TDZ, shoot organogenesis was accompanied with extensive callus formation at the explant surface.

Addition of IAA (0.5 mg/l) in the medium already containing BAP or TDZ, making different combinations, significantly lowered the percentage of response (Table 2). A decrease in the number of shoot buds/shoots per explant, after addition of IAA to different concentrations of BAP, was also observed except for the combination of 0.5 mg/l IAA with 1.0 mg/l BAP which induced considerably higher number of shoots (7.27) per explant, though the percentage of response was low (43.33 %). Addition of IAA to different concentrations of TDZ induced a peculiar type of response, considerably higher number of shoot buds (7.07–12.23) were observed per explant but these shoot buds could not differentiate into full grown shoots.

**Fig. 4:**
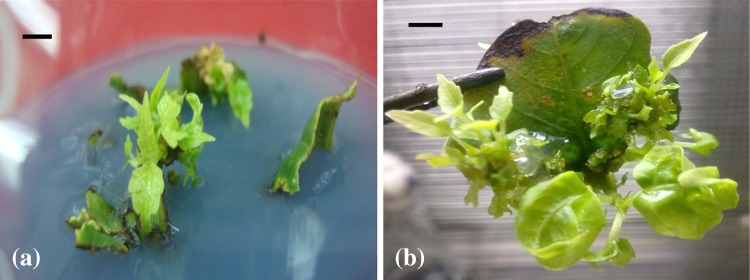
Shoot organogenesis from middle section of the leaf in vertical orientation, when cultured on MS medium having 5.0 mg/l BAP; **a** shoot buds as observed after 28 days of culture, *bar* 0.6 cm; **b** shoots as observed after 60 days of initial culture, *bar* 0.5 cm

Of the thirty-two PGR regimes screened, 5.0 mg/l BAP, besides supporting maximum percent response (100 %), induced proliferation of maximum number of shoot buds (5.83) (as observed after 4 weeks of culture) (Fig. 4a) which later on turned into maximum number of shoots (7.93) with higher lengths (2.97 cm, on an average) (as observed after 60 days of initial culture) (Fig. 4b) (Table 2). Comparable number of shoots (7.27) was obtained on combination of 0.5 mg/l IAA with 1.0 mg/l BAP, but as percentage of response on this particular combination was very low (43.33 %), this was not selected for further studies.

### Shoot multiplication during sub-culturing

**Fig. 5:**
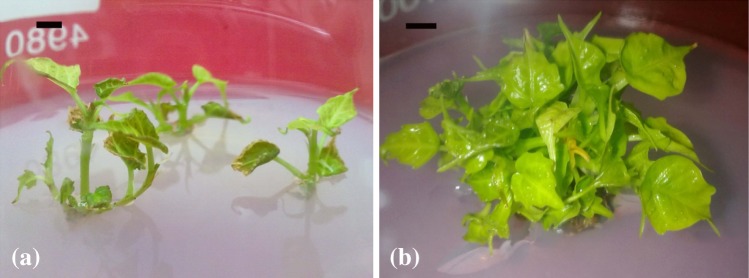
Shoots multiplication during sub-culturing on; **a** MS medium having 5.0 mg/l BAP, *bar* 0.7 cm; **b** MS medium having 5.0 mg/l BAP with additional supplementation of 200 mg/l glutamine, 150 mg/l adenine sulphate and 100 mg/l phloroglucinol, *bar* 0.5 cm

The shoots so obtained, when excised from the explant and cultured in a cluster of 2–3 shoots on the most suitable medium obtained above i.e. MS medium having 5.0 mg/l BAP (control), mild chlorosis of leaves and stem as well as curling and browning of leaves edges and early leaf fall (at low frequency) was observed (Fig. 5a). Further, the number of shoots obtained on this medium, at the end of fifth week, was also less (6.53) (Table 3). Lowering of BAP concentration to 2.5 and 1.0 mg/l further lowered the multiplication rate while health of shoots remained poor (Table 3). To improve quality of shoots as well as to enhance shoot multiplication rate, three additives were added in optimal concentration (200 mg/l glutamine+ 150 mg/l ADS+ 100 mg/l phloroglucinol); optimisation of these additives being discussed in our previous report (Siwach and Gill 2011). Supplementation of these additives to each of the three concentration of BAP significantly increased the shoot multiplication rate as well as improved the quality of the shoots (Table 3). Of the three concentrations of BAP, significantly higher number of shoots (24.37) was obtained when this combination of glutamine, ADS and phloroglucinol was supplemented to 5.0 mg/l BAP (Fig. 5b) indicating the suitability of higher cytokinin concentration even during repeated sub-culturing. The shoots so obtained were isolated after 5 weeks of culture and were sub-cultured on this medium and the process was repeated 4–5 times, before subjecting the individual shoot to rooting.

### In vitro rooting, acclimatisation and transplantation

**Fig. 6:**
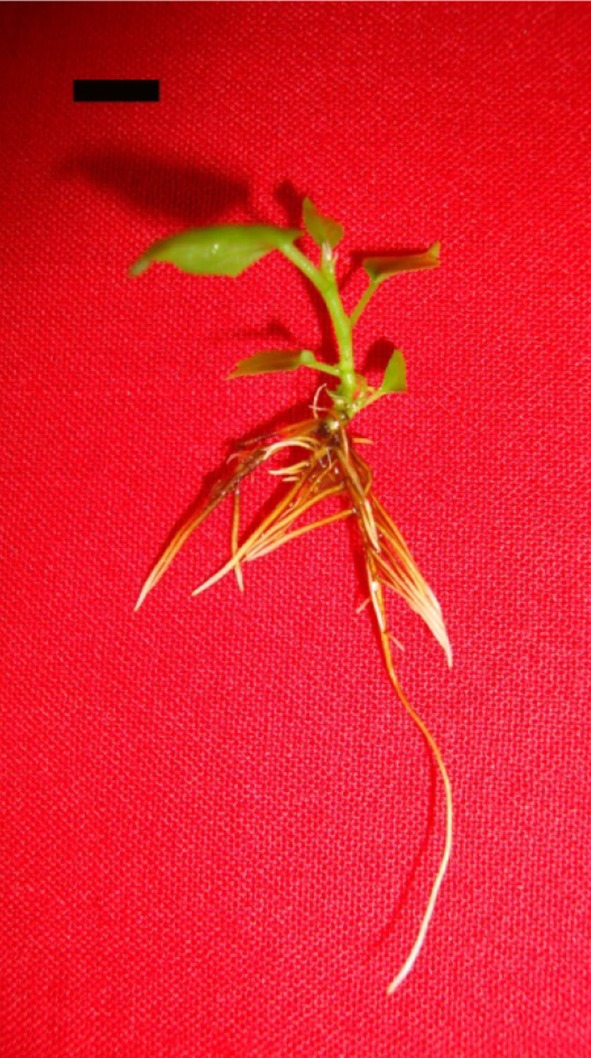
In vitro rooting of micro-shoots obtained on semi-solid MS medium supplemented with 2.0 mg/l IBA and 0.1 mg/l IAA, *bar* 1.0 cm

**Fig. 7:**
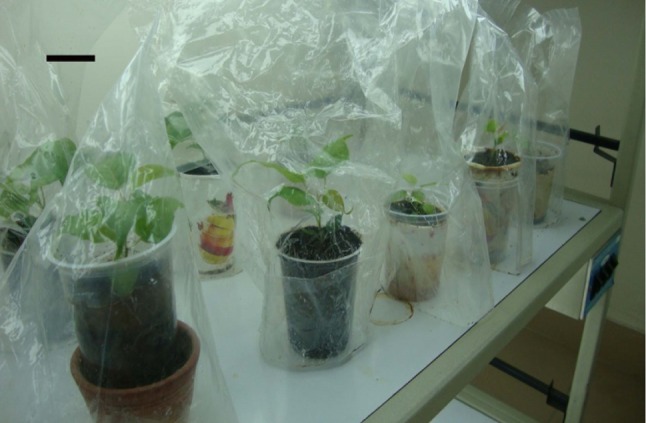
Acclimatisation of transplanted plants under culture room conditions, *bar* 5 cm

**Fig. 8:**
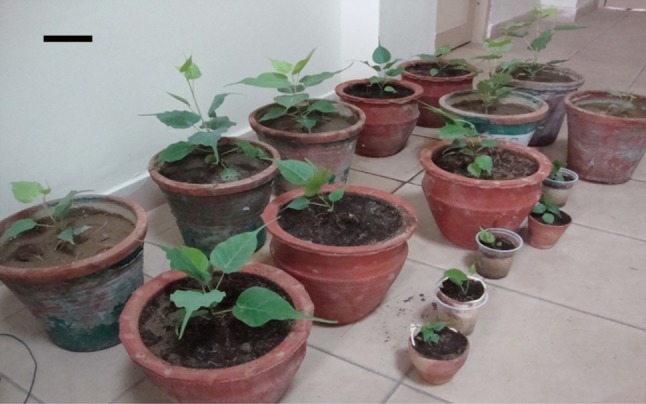
Successful acclimatised, potted plants of different age, *bar* 10 cm

The efficient in vitro rooting of the shoots, regenerated above, was obtained on MS medium supplemented with 2.0 mg/l indole butyric acid (IBA) along with 0.1 mg/l IAA, with a frequency of 95 %, as discussed in earlier report (Siwach and Gill 2011) (Fig. 6). The plantlets were successfully acclimatised (Fig. 7) and transferred to field conditions (Fig. 8) with a survival rate of 90 % on conditions reported earlier (Siwach and Gill 2011).

### Checking the clonal fidelity of micropropagated plants

Out of 20 RAPD primers, amplification with reproducible band pattern was obtained with 13 (primers 1, 2, 3, 4, 9, 11, 12, 13, 14, 17, 18, 19, 20); rest seven primers either did not yield amplification or gave poor non-reproducible band pattern (Table 4). With these thirteen primers monomorphic band pattern was observed in the six randomly selected micropropagated plants and the MP sample, which confirmed the clonal nature of micropropagated plants (Table 4).

**Fig. 9:**
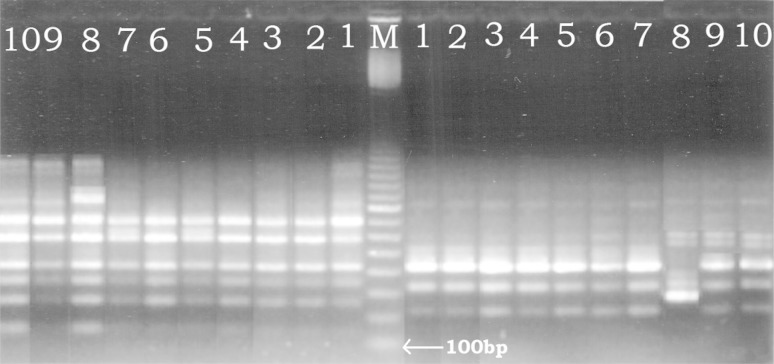
A representative RAPD reaction for checking the clonal fidelity of micropropagated plants and seedling. M: 100 bp ladder (GE Healthcare Life Sciences), Samples on the right of the ladder were amplified with primer 9 (GGGTTTCGCC) and samples on the left of ladder were amplified using primer 11 (TATTCGGCGT). *Lane 1* mother plant (MP); *lane 2–7* micropropagated plants of 1, 2, 4, 5, 6, 7 months age respectively; *lane 8* seedling of 6 months age (Se-1_6M_); *lane 9* seedling of 4 months age (Se-2_4M_); *lane 10* another seedling of 4 months age (Se-3_4M_)

The selected three seedlings exhibited monomorphic band profile among themselves as well as the MP and selected micropropagated plants, when amplified with nine primers-3, 4, 12, 13, 14, 17, 18, 19 and 20 (Table 4). With rest four primers (1, 2, 9, 11), the seedlings yielded different band amplification profile. With primer 1, Se-2_4M_ yielded monomorphic band pattern with MP (single band of 500 bp), while one polymorphic band of 670 and 600 bp was observed for Se-1_6M_ and Se-3_4M_ respectively. With primer 2, a monomorphic band of 700 bp was observed in all the three seedlings (monomorphic with MP and microproagated plants also), while polymorphic band of 840 bp was observed for Se-1_6M_. The MP and micropropagate plants also yielded a band of 1,200 bp with primer 2 and this band was absent in all the three seedlings. The amplification with primer 9 yielded 3 monomorphic bands of 240, 340, 440 bp size in MP, micropropagated plants, Se-2_4M_, and Se-3_4M_, while two polymorphic bands of 590 and 610 bp size were found only in the three seedlings, while a polymorphic band of 300 bp was observed only in Se-1_6M_ (Fig. 9). Likewise, with primer 11, monorphic bands of sizes 350, 410, 600, 700 bp were found in all the ten samples, polymorphic band of 150 bp was found in Se-1_6M_ and Se-3_4M_ and polymorphic band of 900 was observed only in Se-1_6M_ (Fig. 9).

### AChE inhibitory assay

**Fig. 10:**
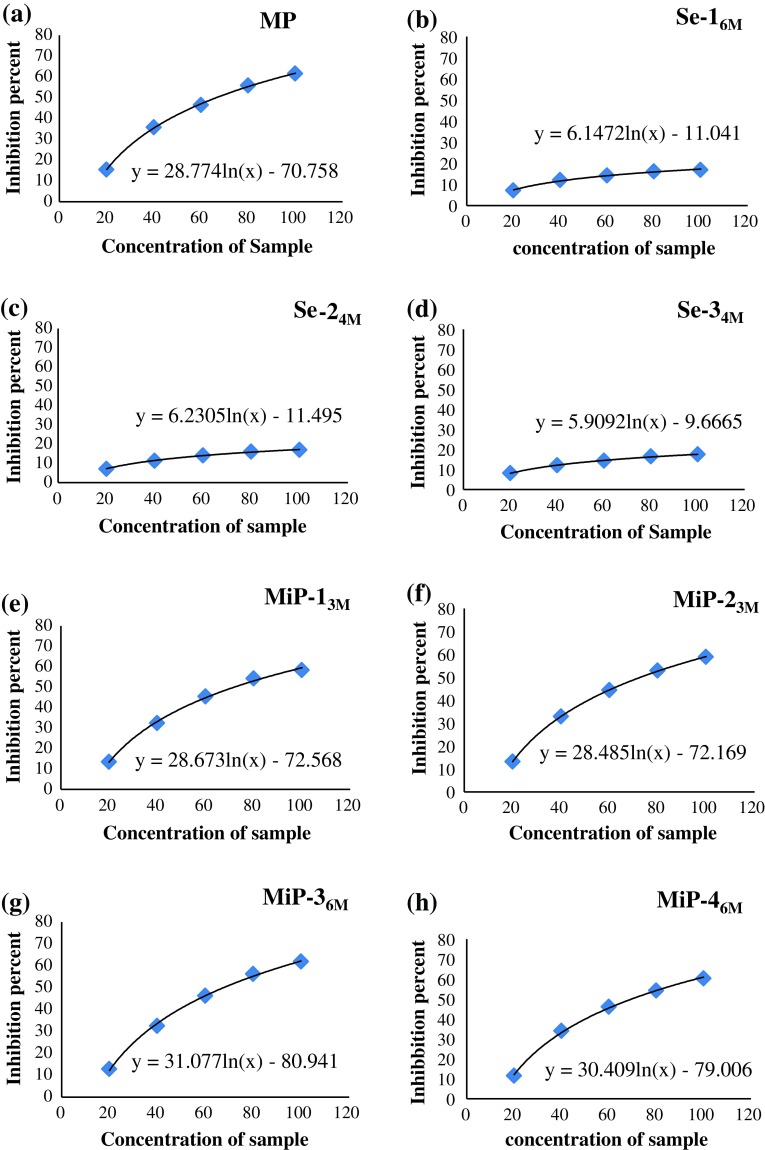
Logarithmic curves representing the percentage of inhibition of AChE enzyme by respective concentrations of different samples viz. **a** MP, **b** Se-1_6M_, **c** Se-2_4M_, **d** Se-3_4M_, **e** MiP-1_3M_, **f** MiP-2_3M_, **g** MiP-3_6M_, **h** MiP-4_6M_

Different concentration of the extracts, of the selected eight samples (MP, Se-1_6M_, Se-2_4M_, Se-3_4M_, MiP-1_3M_, MiP-2_3M_, MiP-3_6M_ and MiP-4_6M_) inhibited the AChE enzyme by different degree, as revealed by AChE inhibitory assay (Fig. 10). The inhibitions obtained with different samples could be categorised in three groups: low (5–20 %), moderate (20–50 %) and good (50–100 %) inhibition. MP exhibited low inhibition at low concentrations of 20 μg/ml (15.35 %), moderate inhibition at 40 and 60 μg/ml (35.75 and 46.48 %, respectively), while at concentration of 80 and 100 μg/ml, good inhibition of 55.80 and 61.58 %, respectively, was observed (Fig. 10a). The IC_50_ value obtained with the mother plant sample was 66.49 μg/ml (Table 5). The 6 months’ aged seedlings (Se-1_6M_) exhibited very poor ability (<20 %) to inhibit AChE enzyme at all the concentrations; the highest concentration of 100 μg/ml, could inhibit the AChE enzyme by only 16.86 % (Fig. 10b). The IC_50_ value obtained for Se was very high (20,538.30 μg/ml) (Table 5). Similar observations were made for two other seedlings of 4 months age; IC_50_ for Se-2_4M_ and Se-3_4M_ being 19,341.03 and 24,281.70 μg/ml, respectively. Contrary to the seedlings, the plants obtained by micropropagation, showed very effective AChE inhibitory activity. At each concentration, the methanolic extracts of all the four selected micropropagated plants exhibited AChE inhibitory activity quite similar to that obtained with MP; good inhibition (more than 50 %) was observed at concentrations of 80 and 100 μg/ml (Fig. 10c–f). In fact, the four selected micropropagated plants, MiP-1_3M_, MiP-2_3M_, MiP-3_6M_ and MiP-4_6M_ exhibited IC_50_ values of 71.87, 72.91, 67.65 and 69.65 μg/ml, which was quite similar to that obtained with MP (66.49 μg/ml) (Table 5).

**Table 5:** IC_50_ values of mother plant (MP), three randomly selected seedlings (Se) and four randomly selected micropropagated plants (MiP) of *F*. *religiosa*, as calculated by the acetylcholinesterase inhibitory (AChEI) activity assay

	Sample	IC_50_ value* (μg/ml)
1	MP (mother plant)	66.49 ± 01.39
2	Se-1_6M_ (of 6 months age)	20,538.46 ± 1.22
3	Se-2_4M_ (of 4 months age)	19,341.03 ± 0.47
4	Se-3_4M_ (of 4 months age)	24,281.70 ± 0.32
5	MiP-1_3M_ (of 3 months age)	71.87 ± 01.28
6	MiP-2_3M_ (of 3 months age)	72.91 ± 00.12
7	MiP-3_6M_ (of 6 months age)	67.65 ± 00.41
8	MiP-4_6M_ (of 6 months age)	69.65 ± 00.42

*Values are means from 3 repeat experiments (each experiment further having three replicates) ± SE

## Discussions

A highly efficient, season-independent, micropropagation protocol has been developed for *F*. *religiosa* L. using leaf explants, followed by comparative AChE inhibitory activity assay of the in vitro raised and conventionally grown plants, for the first time during the present study.

A significant effect of nature and orientation of leaf segments was observed on direct shoot organogenesis; middle section of leaves with cut end inserted inside the medium was found most suitable (Table 1). The orientation of leaf explant has also been reported to play an important role in the micropropagation of some other plants like Marion Blackberry (Meng et al. 2004). In vitro response of explants from woody perennials is reported to be strongly influenced by season of explant collection (Debergh and Maene 1984; Debergh and Read 1991). Such an observation has also been reported for the nodal explants of *F*. *religiosa* (Siwach et al. 2011). However, during the present study, the percentage of response as well as average number of shoots buds per leaf explant was found nearly consistent (with non-significant differences) during all the 12 months of the year (Fig. 3). Such report, mentioning leaves as explants with no seasonal constraints, did not exist previously for any other plant species. One of the possible explanations is that the axillary and apical meristematic cells, of the perennial trees, are genetically directed to divide actively during the active growth season, and this periodic growth direction may not be there with the differentiated tissues like leaves, though to conclude this fact, more studies are needed.

The optimal PGR requirement for direct shoot regeneration from nodal segments of *F*. *religiosa* has been found different by different groups. Deshpande et al. (1998) reported 5 mg/l BAP as suitable for initial bud break and 1.0 mg/l BAP as for further shoot proliferation, Hassan et al. (2009) found 0.5 mg/l BAP in combination with 0.1 mg/l IAA as most suitable while Siwach and Gill (2011) found combination of 1.0 mg/l BAP and 0.5 mg/l IAA best for shoot induction and multiplication. During the present study, with leaves’ segments as explants, a different PGR requirement was observed for direct shoot regeneration. Of the two cytokinins, BAP was found better than TDZ for shoot induction and proliferation (Table 2). Amongst various concentrations of BAP, alone as well as in combination with 0.5 mg/l IAA, used during the present study, BAP at 5.0 mg/l was found most suitable for percentage of response (100 %) as well as for inducing maximum number of shoots per explant (7.93) (Table 2). The suitability of higher level of BAP for shoot induction and proliferation from the leaf explant has also been documented for *Pipernigram* (Ahmad et al. 2010) and *Brassicarapa* (Abbasi et al. 2011). Though combination of 1.0 mg/l BAP and 0.5 mg/l IAA also induced considerably high number of shoots (7.27), it could not be considered as suitable medium because of poor response (43.33 %). Induction of high number of shoots on this particular medium could be attributed to low cytokinin to auxin ratio but the reason for poor response on it could not be explained with present data and literature available. The low cytokinin to auxin ratio has also been reported earlier as the most suitable ratio for efficient shoot induction from the nodal explants of *F*. *religiosa* (Hassan et al. 2009; Siwach and Gill 2011).

Addition of IAA in the medium having BAP or TDZ was found inhibitory for percentage of response. Similar findings of inhibitory effect of combination of auxin and cytokinin for shoot organogenesis frequency have also been reported for some other plant species (Ahmad et al. 2010; Abbasi et al. 2011). Combination of IAA and TDZ was found very efficient in initial shoot bud induction, however, these buds could not grow into shoots and remained stunted. Similar observation was observed with TDZ alone in the medium, during the direct shoot organogenesis from nodal explants of *F*. *religiosa* (Siwach and Gill 2011). TDZ is a substituted phenyl urea (*N*-phenyl-1,2,3-thidiazol-5-yl urea) that has immense potential as a cytokinin in shoot organogenesis in a large number of plant systems, especially in the woody species (Mansouri and Preece 2009). On the other hand, some studies have reported the negative impact of TDZ on shoot proliferation (Feng et al. 2010), however how this effect was further augmented by addition of 0.5 mg/l IAA during the present study could not be explained by the existing literature.

The shoots when excised from the explant and sub-cultured on same medium (BAP 5.0 mg/l) exhibited chlorosis of stem and leaves, curling and browning of leaves edges as well as early leaf fall (Fig. 5a). Such observations were also made during the shoot proliferation from nodal segments of *F. religiosa* (Siwach and Gill 2011) as well as for some other plant species (Husain et al. 2008). The suitability of a particular combination of three additives (200 mg/l glutamine+ 150 mg/l ADS+ 100 mg/l phloroglucinol) for overcoming these problems has been reported and discussed well earlier (Siwach and Gill 2011). During present study, higher concentration of BAP (5.0 mg/l) was also found supportive for shoot multiplication during repeated sub-culturing, as compared to lower one (Table 3). This was contrary to a general observation where cytokinin, at high concentration, favours shoot induction while lowering of concentration is optimal for subsequent shoot proliferation (Deshpande et al. 1998).

The protocol so developed as discussed above is different and more efficient from the existing protocols for micropropagation of *F*. *religiosa*. Jaiswal and Narayan (1985) have reported shoot regeneration via indirect organogenesis through callus phase initiated from nodal explants of *F*. *religiosa*, maximum number of 6–10 shoots being obtained from a callus piece. Likewise Narayan and Jaiswal (1986) have reported differentiation of plantlets, 5–10 in number, from leaf callus of *F*. *religiosa*. Micropropagation via the callus phase has chances of somaclonal variations and so cannot be said to be clonal propagation. Direct shoot regeneration in *F.religiosa* L. has been reported by Deshpande et al. (1998) and in their study, maximum of 5–6 shoots were obtained from a single explant which may be considered as low regeneration rate for a commercially efficient and economically viable micropropagation system. Further no observation has been made for shoot multiplication during repeated sub-culturing, no mention of any observation/problem common to tissue culture of higher age woody perennials, has been made. Hassan et al. (2009) have reported a comparative high rate of multiplication (10–15 shoots per explant) from nodal explants of *F*. *religiosa* but still the study lacked the observations regarding health of the shoots during continuous sub-culturing process. An efficient micropropagation protocol for *F*. *religiosa*, using nodal segments as explants (giving 35 shoots per explant) was reported by us earlier (Siwach and Gill 2011) but culture initiation part had to be restricted to a particular time-period of the year as the response of nodal segments was found to be strongly affected by the season of explant collection (Siwach et al. 2011). The present study observed no effect of season on the in vitro response of mature leaves segments of *F*. *religiosa* and hence leaves were concluded as suitable explants for obtaining season independent micropropagation protocol. Leaves have been taken as explants for micropropagation of many other woody plant species like *Prunusserotina* (Liu and Pijut 2008), *Elaeocarpusrobustus* (Rahman et al. 2003) but weather the response is affected by the season of leaf explant collection has not been studied. Such a finding will be of great importance for developing commercial micropropagation protocol of woody perennials.

The RAPD molecular marker analysis was found successful for checking the genetic fidelity of plants, during the present study. Out of twenty primers, five primers (primers 5, 6, 7, 15, 16) resulted in no amplification in all the samples probably because of lack of formation of stable primer template structure during PCR reaction. Two primers (8 and 10) did not yield stable and consistent amplification profile for any of the selected samples indicating towards the redundancy of complementary sites for these two primers. The 13 primers (primers 1, 2, 3, 4, 9, 11, 12, 13, 14, 17, 18, 19, 20), which resulted in stable amplification in all the samples, exhibited monomorphism among the selected micropropagated plants and MP confirming the clonal nature of these plants. Out of these 13, amplification with four primers (1, 2, 9, 11), exhibited polymorphism among seedlings and MP. Such an observation supports the occurrence of genetic changes during gamete formation and so confirms the propagation via seeds as non-clonal way of propagation. RAPD markers have also been used successfully to assess genetic stability among micropropagated plants of a number of species earlier e.g. *Ribes nigrum* L. (Khan and Spoor 2001).

The 45–50 years old *F*. *religiosa* tree, used as mother plant during the present study was found to possess higher AChE inhibitory activity (IC_50_ of 66.49 μg/ml) than that reported for *F*. *religiosa* (IC_50_ of 73.69 μg/ml) by Vinutha et al. (2007). It can be attributed to the difference in the genotype, physiological state as well as environmental conditions between the samples of the two studies.

The micropropagated plants of 3 and 6 months age were found to exhibit AChE inhibitory activity quite similar to that of mother plant of 45–50 years age (Fig. 10). Contrarily, seedling of the 6 months age and 4 months age exhibited very poor ability to inhibit AChE enzyme. The normal course of development observed with perennial trees include the nil or poor production of secondary metabolites during primary years of growth and development and generally after 20–25 years of growth, secondary metabolites synthesis is accelerated leading to higher level of these metabolites in different parts of the tree (Kulkarni 2000). This could explain the huge difference in the AChE inhibitory activity of the higher age MP (IC_50_-66.49 μg/ml), 6 months old seedling Se-1_6m_ (20,538.30 μg/ml) and 4 months old seedlings—Se-2_4M_ (19,341.03 μg/ml), Se-3_4M_ (24,281.70 μg/ml). The explants, when isolated from the higher age plants, have secondary metabolic pathways genes in the active mode (i.e. switched on state) and these genes generally remain in same state in the plants, regenerated from these explants (Kulkarni 2000). So, in vitro raised plants (from explants of higher age plant) start producing secondary metabolites much earlier than the plants obtained by seed germination and are better source for pharmaceutical application (Gurel et al. 2011; Rao et al. 2011; Jiang et al. 2012). This is the possible explanation of obtaining higher AChE inhibitory activity in the methanolic extracts of 3 and 6 months aged micropropagated plants, compared to that in the 4 and 6 months old seedling. Such observation is of great significance for exploiting the pharmaceutical applications of *F. religiosa* towards AD treatment as it rules out the need of waiting for so many years to let the plant start secondary metabolites synthesis and hence avoids the issues of social, environmental and religious concerns and gives an alternative for large scale commercial production.

## Conclusion

In conclusion, our studies demonstrated that the leaves explant can be a better alternative to apical or nodal explants for developing a season independent regeneration protocol. Higher concentration of BAP (5.0 mg/l) was most suitable for direct shoot regeneration as well as shoot multiplication. During repeated sub-culturing, the medium needed to be additionally supplemented with ADS (150 mg/l), glutamine (200 mg/l) and phloroglucinol (100 mg/l) to overcome the problems of chlorosis and leaf fall. The micropropagated plants were genetically stable as was revealed by RAPD analysis of randomly selected plants of different age. Acetylcholinesterase inhibitory (AChEI) activity of micropropagated plants was quite effective, similar to that of mother plant while 6 months old seedling performed very poorly towards enzyme inhibition. The findings of present study give an alternative source for exploiting AChEI activity at large scale and will be of great use in developing new solutions to Alzheimer disease.

## Abbreviations

BAP: Benzylaminopurine
IAA: Indole acetic acid
TDZ: Thidiazuron
ADS: Adenine sulphate
PGR: Plant growth regulator
AChE: Acetylcholinesterase
AD: Alzheimer disease
MiP: Micropropagated plant
MP: Mother plant
Se: Seedling
RAPD: Randomly amplified polymorphic DNA
